# Brain tissue banking for stem cells for our future

**DOI:** 10.1038/srep39394

**Published:** 2016-12-19

**Authors:** Emily Palmero, Sheryl Palmero, Wayne Murrell

**Affiliations:** 1 Vilhelm Magnus Laboratory for Neurosurgical Research, Institute for Surgical Research, Oslo University Hospital, Oslo, Norway

## Abstract

In our lab we study neurogenesis and the development of brain tumors. We work towards treatment strategies for glioblastoma and towards using autologous neural stem cells for tissue regeneration strategies for brain damage and neurodegenerative disorders. It has been our policy to try to establish living cell cultures from all human biopsy material that we obtain. We hypothesized that small pieces of brain tissue could be cryopreserved and that live neural stem cells could be recovered at a later time. DMSO has been shown to possess a remarkable ability to diffuse through cell membranes and pass into cell interiors. Its chemical properties prevent the formation of damaging ice crystals thus allowing cell storage at or below −180 C. We report here a protocol for successful freezing of small pieces of tissue derived from human brain and human brain tumours. Virtually all specimens could be successfully revived. Assays of phenotype and behaviour show that the cell cultures derived were equivalent to those cultures previously derived from fresh tissue.

The adult human brain contains stem cells that can differentiate into mature neurons that generate action potentials^1^,^2^ and communicate by synapses^3^. It is expected that these cells may be used to treat neurodegenerative diseases (e.g. Parkinson´s disease) and brain injuries as knowledge of their biology progresses. In our lab we study both neurogenesis and the development of brain tumors. Thus we have also worked towards treatment strategies for glioblastoma^4^,^5^,^6^,^7^. It has been our policy to try to establish living cell cultures from all human biopsy material that we obtain in order to pursue these goals^8^,^9^. Subsequent to successful culture, aliquots of neural or tumour stem cells are cryopreserved in a liquid nitrogen cell tank.

In 2013 we published a comprehensive study of neural stem cells derived from human brain biopsies^9^. Types of tissue samples were subventricular zone (SVZ), Hippocampus (HPC), Cortex (Grey and White Matter), Grey Matter (GM), and White Matter (WM) The stem cell we described has the ability to generate vast numbers of cells, differentiate into neurons, astrocytes and oligodendrocytes. As well we established its potential to cross into other lineages by transplanting into the early chick embryo. Cells that normally make neural cell types were also able to make cardiac muscle cells and skeletal muscle cells. This potentiality could be discerned in clones (single cells) and we were able to infer percentage presence of primitive cells per different regions assayed. Assessment of phenotype of the various populations was undertaken using immunochemistry, microarray, proteomics and western blot. Thus information was acquired at transcript and protein level. Having explored a number of alternative culturing conditions we were able to present an efficient method for the establishment and propagation of human brain stem cells from whatever brain tissue samples we tried. We showed virtually unlimited expansion of an authentic stem cell phenotype. Pluripotency proteins Sox2 and Oct4 were expressed without artificial induction. For the first time multipotency of adult human brain-derived stem cells was demonstrated beyond tissue boundaries. Whilst clarification of these cells’ behavior has been ongoing, we hope our work will lead to the future repair of tissues by transplantation of an adult patient’s own-derived stem cells.

Neurosurgical operations can occur at any and unpredictable times. As well there may be heavy workloads if this policy of culture is to be pursued. So availability of suitably trained staff, of reagents and of storage facilities all become management issues.

We were intrigued by futuristic suggestions about preservation of human bodies with the intention of eventual revival one day. A protocol for this was published in 1969^10^ although no humans have yet been brought back! In 1979, Clafrin and Malinin reported cryopreservation of small pieces of tissue from various human organs and subsequent thawing and culture of live cells^11^. There have been suggestions in ‘blogs’ on the internet that such procedures will yield live stem cells but to our knowledge no testing of this has been reported. We hypothesized that small pieces of brain tissue could be cryopreserved and that live neural stem cells could be recovered at a later time. Thus the scientific establishment of such a procedure as this has been the main focus of the work reported here. It is hoped that our work will therefore enable many biologists in their efforts.

Cell storage today commonly uses dimethyl sulphoxide (DMSO) in the protocols published and indeed DMSO figured in the protocol for human body storage mentioned above that was undertaken by Darwin in 1969. DMSO has gained both fame and notoriety in medical science and even health food shops sell it in the USA. Fraudulent claims of its efficacy as a drug have been investigated. DMSO has been shown to possess a remarkable ability to diffuse through cell membranes and pass into cell interiors. Its chemical properties prevent the formation of damaging ice crystals thus allowing cell storage at or below −180 C.

We report here a protocol for successful freezing of small pieces of tissue derived from human brain and human brain tumours (see Supplementary Information). Virtually all specimens could be successfully revived. Assays of phenotype and behaviour show that the cell cultures derived were equivalent to those cultures previously derived from fresh tissue^9^.

## Results

The types of biopsy investigated were tumour Biopsy, subventricular zone (SVZ), Hippocampus (HPC), Cortex (Grey and White Matter), Grey Matter (GM), White Matter (WM) and Ultrasonic Aspirate (UA) of resection sites.

Biopsies to be cryogenically frozen were able to vary in size between 100 mm^3^ down to 8 mm^3^. 100 mm^3^ (100 μL) was the maximum quantity able to be stored in a 1 mL cryotube containing 100 μL DMSO and the remaining volume to a total of 1 mL with serum and thence subsequently be able to produce viable cells efficiently. A tissue volume of 8 mm^3^ was however adequate for establishing robust cultures.

**Figure 1:**
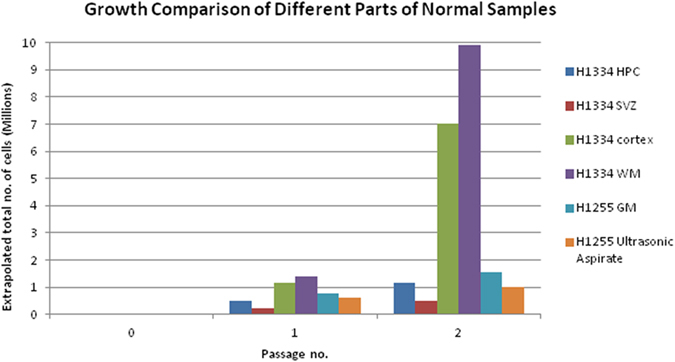
Various tissue sources from ‘normal’ human brain that were cryogenically stored according to our protocol and then later thawed and revived. Cell numbers yielded and proliferative ability differed with source tissue. HPC, hippocampus; SVZ, subventricular zone; Cortex; WM, white matter; GM, grey matter; Ultrasonic Aspirate. Cells described in this figure were grown in *Failsafe* medium. Passage numbers: 0, initial plating; 1, after about three weeks; 2, after about six weeks.

Eight cubic millimeters (8 mm^3^) of freshly cultured normal brain tissue was dissociated enzymatically (viability 85%) and yielded approximately 1.2 million cells. These were plated at 200,000 live cells per mL of culture medium. In five weeks 1,000,000 cells had expanded to 10,000,000 cells (Fig. 1, Murrell *et al*.^9^) but this varied with tissue source (HPC yielding the most). Tumour cultures (usually Glioblastoma) varied according to patient. Cells were cultured in *Failsafe* medium and *VML* medium (*VML* medium promoted growth as neurospheres whilst *Failsafe* resulted in adherent cultures).

### 97% of frozen biopsies were able to be resurrected

**Figure 2:**
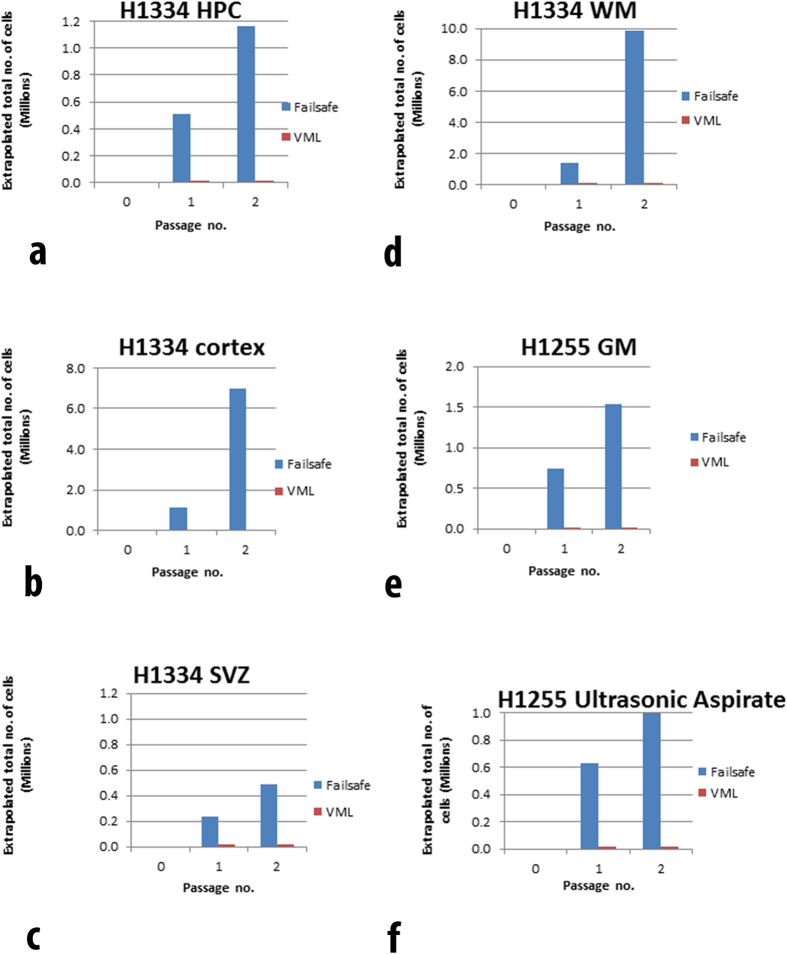
Comparison of growth characteristics of normal brain derived frozen samples using the two media formulations: *Failsafe* and *VML*. The Y axis plots yield of cells (millions). The X axis shows passage numbers: 0, initial plating; 1, replating at about three weeks; 2, replating at about six weeks.

**Figure 3:**
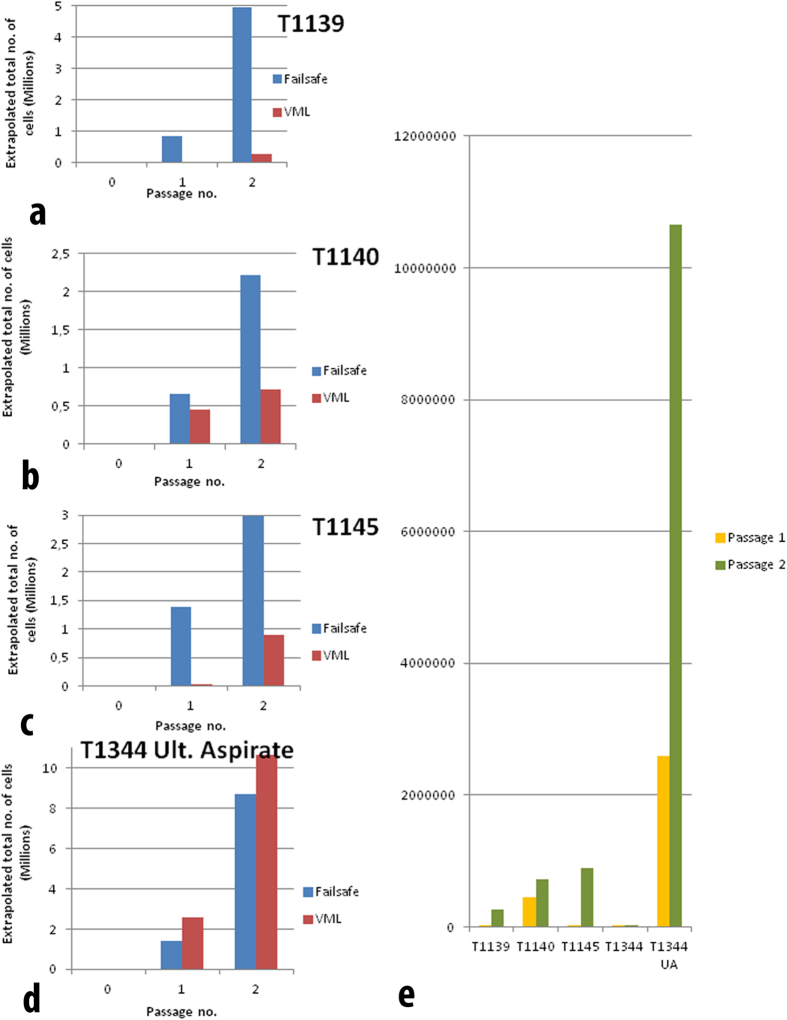
(**a–d**), Four frozen patient tumour samples thawed and grown with the two media formulations: *Failsafe* and *VML*. In the case of tumour biopsies, *Failsafe* yielded better numbers than VML usually. In the case of ultrasonic aspirate derived tissue fragments, *VML* medium produced very rapid expansion of numbers. *VML* is used to promote growth as spheroids but in this case T1344UA grew as an adherent culture. Remarkably the T1344 tissue biopsy yielded no viable cells (**e**). The Y axis plots yield of cells (millions). The X axis shows passage numbers: 0, initial plating; 1, replating at about three weeks; 2, replating at about six weeks.

25 chopped biopsies were obtained from tumour tissue and nine samples from ‘normal’ brain tissue. Using the protocol described here, 30 of these 31 stored tissue samples could be revived to produce viable cell cultures using *Failsafe* medium, whilst 26 could be induced to grow cells using the *VML* serum-free culture protocol. When frozen tissue fragments were thawed and washed clear of DMSO, they were slow to yield living cell outgrowths, presumably because they had not been dissociated enzymatically. After a week though their growth was rapid with 1,000,000 cells being attained by three weeks. Again the different sources yielded different numbers of viable cells (Figs 1, 2, 3).

### All normal human brain tissue sources yielded viable cultures. 100% of patients yielded successfully revived viable cells

In the case of the tumour tissue sample that did not yield viable cells, an ultrasonically aspirated efflux of the resection site did yield viable cells in great numbers^12^. So in fact all patient sources were successfully recovered using this method.

The *Failsafe* medium, containing 1% serum and mitogenic growth factors was superior to the *VML* serum free medium in all cases with regard to overall revival success, and produced vastly superior numbers except for one situation.

### Phenotype of the cultures derived from frozen biopsies

Immunohistochemistry was used to determine some aspects of the cultures’ cellular phenotype in order to compare cultures resurrected from frozen pieces of tissue with those grown freshly from biopsies. As well normal brain- and tumour-derived resurrected cultures were compared. Markers used were for stem cells, OCT4; neural stem cells, NES; glia, GFAP; oligodendrocytes, OLIG2 and neurons, TUBB3 and NEFH.

**Figure 4:**
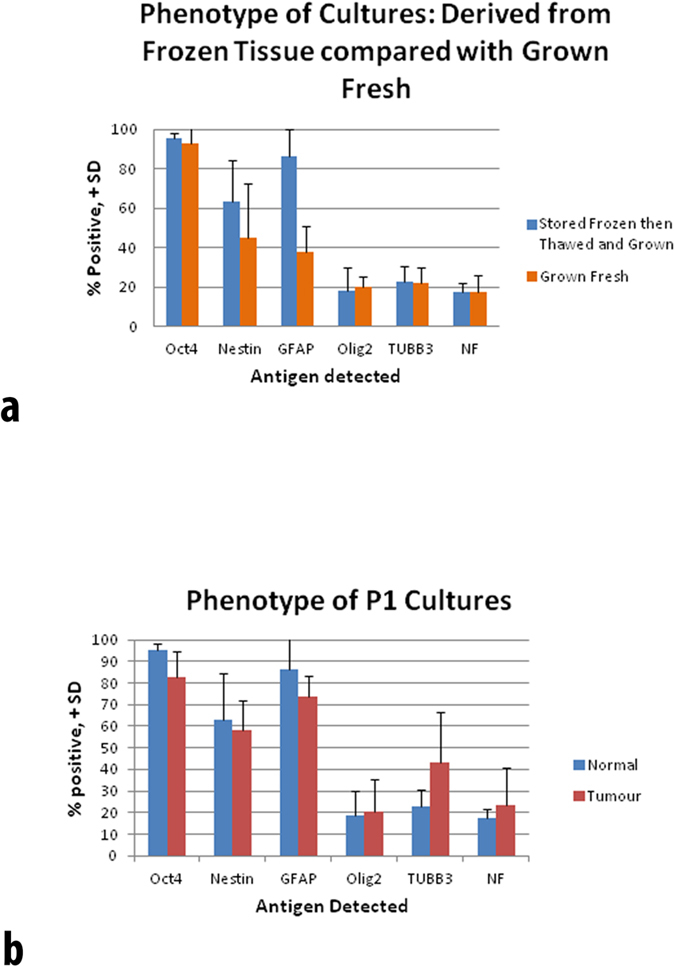
(**a)** Cultures were from normal brain tissue. Comparison of percentage of cells positive for selected phenotype markers in cultures derived from frozen tissue fragments and those derived from fresh biopsies. Cultures only differed in expression of GFAP (p = 0.0029). (**b**) Comparison of percentage of cells positive for selected phenotype markers in cultures derived from frozen normal tissue with cultures derived from frozen tumour tissue samples.

Our earlier studies had shown that no statistically significant phenotypic differences could be discerned between the neural stem cell cultures derived from different source locations within human brain^9^. So in Fig. 4a, data from the multiple sources were averaged for comparison. Frozen and subsequently resurrected cultures were from hippocampus (HPC), white matter (WM), cortex, grey matter (GM) and subventricular zone (SVZ). Cultures freshly derived from the same tissue source types were averaged to compare percentage of cells positive for each marker.

In the case of adherent cell cultures derived from normal brain tissue, the cell populations were comparable with those described by Murrell *et al*.^9^. That is, they appeared to consist of neural stem cells and partially committed progenitors as described therein (Fig. 4a). There was one notable difference when comparing frozen-derived to fresh grown cultures: There was a statistically significant increase in the number of GFAP-positive cells (p = 0.0029, Students T-test, Two-tailed, Type Two, n = 5 for frozen and n = 4 for fresh).

When the phenotypes of normal- and tumour-derived resurrected cell cultures were compared, they had roughly the same proportions of marker-positive cells (Fig. 4b). Tumour-derived cultures are usually similar to neural stem cell cultures in that NES-, GFAP-, OLIG2- and TUBB3-positive cells are all present.

In the case of all three cell culture types: normal human brain-derived, normal human brain frozen biopsy-derived and glioblastoma frozen biopsy-derived, the phenotype was strongly that of neural stem cells. That is there was high percentage positive expression of both OCT4 and NES.

## Discussion

We have successfully derived living stem and progenitor cells from human brain tissue that was frozen according to our protocol. This was achieved in virtually all samples. Since our initial testing of this procedure, more than 120 biopsy specimens have been stored this way. Tissue sources have been various grades of tumour (astroctoma, glioma, glioblastoma) and normal brain tissue described as SVZ, GM, WM, Cortex and hippocampus. It has been the policy of our lab to culture cells from all possible human tissue samples removed by surgeons of OUS’ Neurosurgery Department.

By three weeks a tissue fragment initially 8 cubic mm, cryogenically processed and then recovered many weeks later produced the same number of cells (about a million) as the number of viable cells initially retrieved from a fresh piece of the same tissue.

In the case of adherent cell cultures derived from normal brain tissue, the cell populations were comparable with those described by Murrell and co-workers for freshly grown cultures^9^. That is, they appeared to consist of neural stem cells and partially committed progenitors as described therein (Fig. 4a). There was one notable difference when comparing frozen-derived to fresh grown cultures: There was a statistically significant increase in the number of GFAP-positive cells. Since the proportions of cells in the cultures that were labeled with neuronal and oligodendrocyte markers were not different between the two sources of culture derivation it follows that more cells must be double labeled with GFAP. This implies that the process of tissue freezing and following revival of cells has somehow favoured an increased number of early progenitors that express GFAP. Double labeling of cells for the different neural sub-types in brain-derived cultures has been clearly shown to occur^9^. The contention that the progenitors derived from frozen tissue are more primitive is based on the currently held paradigm that as cells become more differentiated they become more restricted in their phenotypic repertoire. This issue nevertheless should be clarified in future studies.

POU5F1 assessment has been questioned recently as a reliable marker when using antibody detection^13^. We used one antibody for ICC, R&D clone 1759 which used human Oct 4 amino acids (aas) 1 to 265 as the immunogen. As well we did Western Blot^9^ with Cell Signaling mAb 4286. Known isoforms of Oct 4 protein range from 256 to 360 aas. Our Western Blot had one clear band at 46 kDa (the correct size) see^9^. Anyway results were consistent with those published in^9^.

The normal procedure worldwide for handling tumour material post-operatively has been to fix and freeze material for pathology assessment. These methods and storage protocols have precluded the possibility of obtaining living cells for analysis. Assessment of protein and RNA expression from these stored frozen samples has been possible but this was the extent of analyses. In terms of attempting to derive treatments some groups have used this dead tissue as a source of protein lysate to instruct dendritic cells for injection of autologous dendritic cell vaccines (DCVax^14^). Our lab has collaborated in the derivation of mRNA instructed DC vaccines where the mRNA has been derived from living tumour stem cells^4^. As well we have used these living TSCs to provide starting material for microarray analyses that compared TSCs to NSCs resulting in identification of potentially useful therapeutic targets in GBM^5^,^15^. To undertake studies of these TSCs’ and NSCs’ biology the availability of living cells has been an exciting opportunity. We have been able to undertake gene modifications enabling target gene knockdowns, followed by *in vitro* and *in vivo* assessment of tumorigenicity^6^,^7^,^5^. Whilst these exciting experimental opportunities have been enabled by our policy of culturing everything, it is often only later that we receive confirmation of diagnosis. With our new procedure enabling the storage of tissue that will yield live stem cells later, we can decide retrospectively which samples to study.

As well though there are economic incentives for following our freezing of viable tissue. Based on past experience it costs about four times as much (405 USD) for culture to three passages of living cells compared to 108 USD for preservation of tissue with our protocol. There are clearly difficulties in attending to biopsies out of hours, having staff availability, maintaining stored reagents etc. Our successful resurrection of live stem cells has alleviated many such concerns.

Because we have had the opportunity to use live cell cultures in our studies we have been able to use microarray and genomic comparisons between normal and tumour stem cells^5^,^9^,^15^ in order to identify possible gene targets. Following this, we have conducted experiments knocking down such targets with lentiviral vectors transduced into stem cells which have enabled assessment of gene function, relevance to tumorigenicity and so on^5^,^6^,^7^.

It has been a tradition to grow neural and brain tumour stem cells using the neurosphere serum free method first established by Reynolds and Weiss^16^ which we emulate in our *VML* medium. However this medium only produces successful cultures from 80% of glioblastoma samples and is very inefficient and unreliable for culturing normal human brain stem cells^9^. We have modified this medium to contain 1% serum and replaced EGF by TGFα. This medium we call *Failsafe* because it is indeed failsafe. All tumour samples (even low grade) and all normal brain tissue samples yield successful cultures using this medium. The serum used can be that of the patient (autologous) if patient treatment is to be derived from cultures. Cultures can be put from one medium to the other^9^. Sphere cultures can be grown adherently; adherent cultures can be grown as spheres^9^. Adherently grown cultures are tumorigenic in most cases when transplanted to the brains of SCID mice^17^. We hypothesized that small pieces of tissue could be stored in suitable cryo-preservative and later be used as a source to recover live stem cells. Having demonstrated this successfully we now have the capability to freeze tissue fragments first and later initiate the sorts of experiments that we can design at leisure.

The issues of growing and establishing stem cell culturing are diverse and complex. There are many confounding factors that need assessment to authenticate experimental results. Bona fide stemness needs to be regularly demonstrated. Minimum criteria for this is capacity to be cloned and multipotency^9^. As well the issue of phenotype assessment and reliability of methods needs clarity. Specially designed primers for qPCR may resolve some confusion but the occurrence of pseudogene sequences in DNA make this difficult. Western blot using monoclonal antibodies may well be necessary to establish expression of genuine protein markers. Alternative assessments such as facilitated cell sorting and analysis may well isolate subpopulations of cells with differing potential. In the case of tumour stem cells, the potential to demonstrate susceptibility to designer drugs or to supply gene information for the construction of personalized dendritic cell-based vaccines provide good motivation for these efforts. The same can be said for tissue-derived autologous stem cells and their potential for tissue regeneration technology. These future prospects make the protocol published here even more relevant and exciting as it gives greater scope and opportunity to think through well designed experiments without the pressure to instantly culture patients’ cells.

## Methods

Ventricular wall biopsies were obtained from temporal lobe specimens removed due to medically refractory epilepsy. Tissue was obtained from consenting patients, ranging in age from 23 to 62 years. Tissue harvesting was approved by the Norwegian National Committee for Medical Research Ethics. Tumour biopsy specimens were obtained from informed and consenting patients, and the tissue harvesting was approved by the Norwegian National Committee for Medical Research Ethics (07321b). Biological material and personal health data has been collected and used in accordance with informed consent from patients included in the study. All experimental procedures were carried out in accordance with the guidelines of the Norwegian National Committee for Medical Research Ethics after being approved by the Regional Ethical Committee (REC South-East S-07321d).

The biopsies were transported from the operating theatre in Leibowitz-15 medium (L15, Invitrogen, Carlsbad, CA), stored at + 4 °C and initially cultured as described previously^1^,^2^. Surgical specimens have been described as the following sources: hippocampus (HPC), subventricular zone (SVZ), white matter (WM), grey matter (GM), cortex, paraventricular zone and mixtures of these. For the most part data presented in this paper emanate from HPC-, SVZ-, WM- and GM-derived cultures only.

### Suspension cell culture from fresh tissue

Tissue samples were minced with a McIlwain Tissue Chopper (Mickle Laboratory Engineering, UK) and digested in 20 units/mL papain (Worthington, NJ) incubated at 37 °C for five minutes. The digestions were stopped by the addition of 10 mL DPBS (Dulbecco’s Phosphate Buffered Saline, Lonza, BioWhittaker) with 1% vol/vol albumin (Octapharma AS). The pellets collected by centrifugation at 300 g for five min at room temperature (RT) were resuspended in DMEM/F12 and counted using a NucleoCounter (Chemometec, Denmark) (to exclude dead cells) and cultured as described previously^1^,^2^. The cells are usually seeded at a density of 10–15,000 cells/cm^2^ ie. 100,000 cells/mL in DMEM/F12 (Invitrogen) supplemented with 2% B27-without retinoic acid (RA, Invitrogen), 10 mM hepes (Lonza, BioWhittaker), 2.5 μg/mL heparin (LeoPharma AS) and 1% penicillin/streptomycin (Lonza, BioWhittaker) in non-treated flasks (Nunc, VWR) at 37 °C in 5% CO_2_.

The cultures were supplemented with 10 ng/mL basic fibroblast growth factor (bFGF, R&D Inc, Minneapolis) and 20 ng/mL epidermal growth factor (EGF, R&D Inc) three times a week that resulted in formation of floating aggregates (neurospheres) and additional DMEM/F12 was added once a week^18^. For subsequent neurosphere formation (cell passage), the neurospheres were dissociated into single cells when they reached 12–15 cells in diameter, by incubating in papain followed by mechanical resuspension and cultured further in the presence of the aforementioned mitogens. This suspension culture method is the standard method used to propagate tumour-derived spheres in our laboratory^8^,^19^,^20^,^21^,^22^ and is called ‘*VML*’ culture protocol.

#### Failsafe medium

The optimal culture medium established by Murrell and coworkers in 2013^9^ was DMEM/F12 with 10 ng/mL bFGF, 20 ng/mL TGFα, 2.5 μg/mL heparin, 2% B27 (without retinoic acid), 10 mM hepes, 1% Pen/Strep and 1% FBS. Cells are usually seeded at 2–4000 cells/cm2 ie. 200000 cells/mL. Cell cultures were kept in a 37 °C incubator with 5% CO_2_. *Failsafe* medium usually results in adherent cell cultures^9^.

#### Types of samples collected were

Tumour Biopsy, subventricular zone (SVZ), Hippocampus (HPC), Cortex (Grey and White Matter), Grey Matter (GM), White Matter (WM) and Ultrasonic Aspirate (UA).

### Cell freezing protocol

Cells in their medium were scraped, poured off and then centrifuged at 300 g for 5 minutes. The supernatant was removed, after which 90% by volume of FBS and 10% by total volume of DMSO (Sigma) were added. FBS could be substituted with autologous patient serum if required. The suspension was transferred to a 1 mL cryotube. The cryotube was stored in a Mr. Frosty (Nalgene) at −70° C in a freezer for a minimum of 4 hours. Then the cryotube was transferred to a liquid nitrogen tank (−196 °C) for long-term storage.

### Sample Collection

Tissue samples from neurosurgical operations were divided into three parts (approximately 8 mm^3^ each) and preserved firstly in one cryotube (Greiner Bio One) for snap-freezing for subsequent protein extraction and stored in a −80 degree freezer. Another cryotube was similarly stored for subsequent RNA extraction. The third sample was placed in Leibovitz L-15 Medium (Lonza, Inc) and transported on ice.

### Tissue dissection

The tissue was placed in a glass bottom dish (WillCo-Dish) with a minimal amount of L-15 (enough to keep the sample from drying). Blood was removed and the sample was cut into small pieces using a #22 Disposable Scalpel (Swann-Morton). Tissue was further dissected using a McIlwain Tissue Chopper (to pieces approximately 0.1 mm^2^). The chopped tissue was collected in L-15 (10 mL) and centrifuged at 300 g for 5 minutes. The supernatant was removed, after which 90% by volume of FBS and 10% by total volume of DMSO (Sigma) were added. The suspension was transferred to a 1 mL cryotube. The cryotube was stored in a Mr. Frosty (Nalgene) at −70° C in a freezer for a minimum of 4 hours. Then the cryotube was transferred to a liquid nitrogen tank (−196 °C) for long-term storage.

### Cell Culture after freezing

The cryotube was collected from the liquid Nitrogen storage tank. The cap was loosened slightly to release pressure and then closed again tightly. The tissue suspension in the cryotube was thawed quickly in a 37°C water-bath. The suspension was still cold when removed. It was transferred to a 15 mL tube. Nine mL of prewarmed culture medium (37°C) was added slowly in droplets and mixed gently. The suspension was centrifuged at 300 g for 5 minutes and supernatant removed. Thus all DMSO was washed from the tissue fragments. The tissue fragments were resuspended in 10 mL of culture medium (37°C) and plated in a 75 cm^2^ flask (NUNC T75-156499). The culture was fed three times a week with 10 ng/ml bFGF (R & D Systems), and 20 ng/ml TGF-α (R & D Systems).

The medium was replaced every 10 days until the cells were ready for passaging (approximately 70% confluent).

### Protocol for immunohistochemical labeling in plastic multiwell plates

Cells were rinsed gently in PBS or HBSS and then fixed in 4% paraformaldehyde (PFA) in PBS pH 7.4, for 10 min at RT. Fixative was washed off with PBS three times. Cells were permeablised and blocked for non-specific binding with 10% serum of the animal the 2° antibody was raised in in PBS pH 7.4, 1% Triton X-100, 0.3% H_2_O_2_ (to quench endogenous peroxidases) for 1 h, at RT, on a shaker. After two washes they were incubated in 1° antibody, 2% serum in PBS, pH 7.4 for 1 hour (h) at RT on a shaker. They were then washed twice in PBS, 0.1% Tween 20, 1% serum, for 10 min, on a shaker. This was followed then by a wash omitting the Tween 20. Biotinylated secondary antibodies (Vector Labs) were then applied for 30 min, at RT in 1% serum in PBS on a shaker. Washing and detection using ABC reagent (avidin-biotin-complex) were performed according to the manufacturer’s instructions (Vector Labs). Plates were stored in 0.1% azide in PBS at 4° C.

#### Antibodies used for immunohistochemical labeling

Rabbit anti-GFAP (glial fibrillary acidic protein), 1/200 (Code No. 20334, DakoCytomation, Denmark); goat anti-OCT 4 (POU5F1), 1/200 (Prod. No. AF1759, R & D Systems); goat anti-OLIG 2, 1/200 (Prod. No. AF2418, R & D Systems); mouse anti-NEFH (Neurofilament 200, NF200), 1/200 (Clone NE14 Prod. No. N5389, Sigma); mouse anti-TUBB3 (βTubulin 3), 1/200 (product number SAB3300047); mouse anti-NES (Nestin), 1/200 (Clone 10C2, Millipore); and mouse anti-P4HB (Prolyl-4-hydroxylase beta subunit), 1/200 (Clone 3-2B12, Acris, USA).

### Controls for immunohistochemistry

Mouse anti-Prolyl-4-hydroxylase beta subunit was used as a positive control for the staining procedure. The target of this antibody acts as either a subunit of Prolyl-4-hydroxylase or as protein disulphide isomerase^23^ and is a ubiquitous antigen in all cells. The remaining antibodies are phenotypic markers and their dilution was determined such that some cells in the cultures were not stained. No primary antibody and no secondary antibody wells were also used for technical controls.

### Statistics

Cultures derived from fresh tissue and cryogenically frozen tissue were compared for comparative phenotypic expression (Fig. 4a). The expression of GFAP differed and was assessed using the Students t-Test (p = 0.0029, Students T-test, Two-tailed, Type Two, n = 5 for frozen and n = 4 for fresh).

## Additional Information

**How to cite this article**: Palmero, E. *et al*. Brain tissue banking for stem cells for our future. *Sci. Rep.* **6**, 39394; doi: 10.1038/srep39394 (2016).

**Publisher's note:** Springer Nature remains neutral with regard to jurisdictional claims in published maps and institutional affiliations.

## Supplementary Material

Supplementary Information
