# Supplementary material for: Brain tissue banking for stem cells for our future
Source: Sci Rep. 2016 Dec 19;6:39394. doi: 10.1038/srep39394 (PMC5171803; doi:10.1038/srep39394)
Supplement: Supplementary Information [file srep39394-s1.pdf]

Brain tissue banking for stem cells for our future

Emily Palmero, Sheryl Palmero and Wayne Murrell

Protocol

Sample Collection

Tissue from the operation is divided into three parts (approximately 2 mm^3 each) and preserved in:

1. Cryotube (Greiner Bio One), snap-freeze (for protein) and store in -80 degrees.
2. Cryotube, snap-freeze (for RNA) and store in -80 degrees.
3. Leibovitz L-15 Medium (Lonza, Inc) and transport on ice (for cryogenic storage of tissue).

Types of Samples Collected

- ✓ Tumour Biopsy
- ✓ SVZ
- ✓ Hippocampus
- ✓ Cortex (Grey and White Matter)
- ✓ Grey Matter
- ✓ White Matter
- ✓ Ultrasonic Aspirate

Tissue Chopping

1. Place the tissue in a glass bottom dish (WillCo-Dish) with a minimal amount of L-15 (enough to keep the sample from drying).
2. Remove blood and cut into small pieces (#22 Disposable Scalpel, Swann-Morton).
3. Dissect further using McIlwain Tissue Chopper (approximately 0. 1 mm^2).
4. Collect the chopped tissue in L-15 (10mL) and centrifuge at 300 g for 5 minutes.
5. Remove supernatant.
6. Add 90% FBS and 10% Dimethyl Sulfoxide by total volume (DMSO, Sigma).
7. Transfer suspension in cryotube (1mL/tube).
8. Store cryotube in Mr. Frosty (Nalgene) at -70°C Freezer (min 4 hours).
9. Transfer the cryotube in the liquid nitrogen tank (-196°C) for long-term storage.

Cell Culture

1. Collect the cryotube from the liquid Nitrogen tank. Slightly loosen the cap of the cells to release pressure. Then close again tightly.
2. Thaw QUICKLY in 37°C water-bath (suspension must still be cold).
3. Transfer suspension to 15 mL tube. Add 9 mL *Failsafe* Culture Medium (37°C) SLOWLY in droplets. Mix gently.
4. Centrifuge at 300 g for 5 minutes
5. Remove supernatant. This removes DMSO from the future culture.

6. Resuspend in 10 mL Culture medium (37°C) and plate in 75 cm^2 flask (NUNC T75-156499).
7. Feed the culture 3x a week with 10 ng/ml bFGF (R & D Systems), 20ng/ml TGF-α (R & D Systems).
8. Replace the medium every 10 days until the cells are ready for passaging (approximately 70% confluent).

Supplementary 1: MATERIALS

Reagents

**DMEM/F12 (1:1)** (1X)	GIBCO, cat. no. 31331-028, with GlutaMAX
**Leibovitz L-15** (1X)	LONZA, cat. no. 12-700F, without L-Glutamine
**HEPES Buffer** (1 M)	LONZA, cat. no. 17-737E
**Penicillin/Streptomycin** (100X)	LONZA, cat. no. 17-602E, 10000 U/mL each<br>1 mL aliquots stored at –20°C.
**bFGF (FGF$_2$)** (10 μg/ml) (Recombinant Human)	R & D Systems, cat. no. 233-FB, 25 μg vial, –20°C<br>Reconstitute in 2.5 mL of PBS containing 1 mM DTT and 0.1% BSA. Aliquot accordingly and store in –20°C.
**EGF** (20 μg/ml)	R & D Systems, cat. no. 236-EG, 200 μg vial, –20°C<br>Reconstitute in 10 mL of 10mM Acetic Acid containing 0.1 % BSA. Aliquot accordingly and store in –20°C.
**TGF-α** (100 μg/ml)	R & D Systems, cat. no. 239-A, 100 μg vial, –20°C<br>Reconstitute in 1 mL Sterile H_2O, Aliquot accordingly and store in –20°C
**B-27** (50X)	GIBCO, cat. no. 12587-010 (<u>without</u> Vit A)<br>1 mL aliquots stored at –20°C.
**Heparin** (0.5 mg/mL)	LEO Pharma AS, cat. no. 585661, 100 IE/mL, +4°C
**Fetal Bovine Serum** (100%)	BioChrome, cat. no. BCHRS0615<br>10 mL aliquots stored at –20°C
**Albumin** (200mg/ml) (Human Serum)	Octapharma, cat. no. 478172, +4°C

**DPBS** (1X)	LONZA, cat. no. 17-512F, Ca^{2+} and Mg^{2+} Free
**DMSO** (100%)	Sigma, D2650
**Papain** (25 U/mg)	Worthington Biochemical Corporation, cat. no. LS003118, 25 mg. Reconstitute in 4.848 mL EDTA (yields 10X Solution) and 100 μL aliquots stored at -20°C. Mix 1 aliquot with 900 μL EDTA to obtain working solution of 13.2 U/mL.
**EDTA** (0.02%, 100 mL)	Sigma, E8008, +4°C
**Trypsin-EDTA** (1X)	GIBCO, cat no: 25300-054, (0.05% EDTA)<br>Aliquots (1 mL) stored at -20°C.

Equipment, plastic ware and other materials

**Strainer**	(70 μm) Falcon, cat. no. 352350
**Flask**	NUNC, T75-156499(Fail-Safe); T75 156800 (Neurosphere)
**Scalpel**	Swann-Morton, #22
**Glass Bottom Dish**	WillCo-Dish®, GWSt-5040, 50/40 mm
**Cryotubes**	Greiner Bio One, 2 mL. Cat. No. 126263
**Glass Pasteur Pipettes**	VWR, Cat. No. 612-1701, 150 mm
**LABOPORT Mini Pump**	KNF Neuberger, Model no. N86KN.18
**Mr. Frosty Freezing Container** (1°C/min)	Nalgene, cat. no. 5100-0001
**NucleoCounter**	Chemometec, NC-100
**Tissue Chopper**	McIlwain, Inc.
**Tissue Culture Hood**	Thermo Electron Corporation, HeraSafe
**CO_2 Incubator**	Thermo Electron Corporation, HeraCell 240
